# Envelope Exchange for the Generation of Live-Attenuated Arenavirus Vaccines

**DOI:** 10.1371/journal.ppat.0020051

**Published:** 2006-06-02

**Authors:** Andreas Bergthaler, Nicolas U Gerber, Doron Merkler, Edit Horvath, Juan Carlos de la Torre, Daniel D Pinschewer

**Affiliations:** 1 Institute of Experimental Immunology, Department of Pathology, University Hospital of Zurich, Zürich, Switzerland; 2 Department of Neuropathology, Georg-August-Universität, Göttingen, Germany; 3 The Scripps Research Institute, Molecular Integrative Neuroscience Department (MIND) IMM-6, La Jolla, California, United States of America; National Institutes of Health, United States of America

## Abstract

Arenaviruses such as Lassa fever virus cause significant mortality in endemic areas and represent potential bioterrorist weapons. The occurrence of arenaviral hemorrhagic fevers is largely confined to Third World countries with a limited medical infrastructure, and therefore live-attenuated vaccines have long been sought as a method of choice for prevention. Yet their rational design and engineering have been thwarted by technical limitations. In addition, viral genes had not been identified that are needed to cause disease but can be deleted or substituted to generate live-attenuated vaccine strains. Lymphocytic choriomeningitis virus, the prototype arenavirus, induces cell-mediated immunity against Lassa fever virus, but its safety for humans is unclear and untested. Using this virus model, we have developed the necessary methodology to efficiently modify arenavirus genomes and have exploited these techniques to identify an arenaviral Achilles' heel suitable for targeting in vaccine design. Reverse genetic exchange of the viral glycoprotein for foreign glycoproteins created attenuated vaccine strains that remained viable although unable to cause disease in infected mice. This phenotype remained stable even after extensive propagation in immunodeficient hosts. Nevertheless, the engineered viruses induced T cell–mediated immunity protecting against overwhelming systemic infection and severe liver disease upon wild-type virus challenge. Protection was established within 3 to 7 d after immunization and lasted for approximately 300 d. The identification of an arenaviral Achilles' heel demonstrates that the reverse genetic engineering of live-attenuated arenavirus vaccines is feasible. Moreover, our findings offer lymphocytic choriomeningitis virus or other arenaviruses expressing foreign glycoproteins as promising live-attenuated arenavirus vaccine candidates.

## Synopsis

Arenaviruses such as Lassa fever virus (LFV) account for substantial mortality in endemic Third World countries and represent potential bioterrorist weapons. Live-attenuated vaccine strains would likely represent an optimal strategy for prevention, but their rational design and engineering have been thwarted by technical limitations. As an additional difficulty, arenaviruses have only four genes, all of which are needed for the infectious cycle. Unlike for other virus families, attempts at deleting a “virulence gene” would therefore interfere with the virus' viability and thereby with its immunogenicity. Using the prototype arenavirus as a model, the authors have developed the necessary tools to investigate an alternative strategy for tailoring of live-attenuated strains: Recombinants were engineered to express a foreign envelope gene instead of the natural one. Thereby, the virus was crippled but still viable. When testing such “envelope-exchange” viruses in a mouse model, they failed to cause disease. Nevertheless, they elicited rapid and long-lived immunity against overwhelming infection and lethal disease upon wild-type virus challenge. This delineates a novel general strategy for the reverse genetic engineering of live-attenuated arenavirus vaccines to be used in endemic areas or in case of a bioterrorist attack.

## Introduction

**Figure 1:**
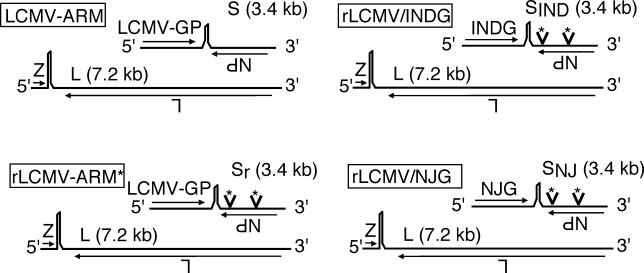
Wild-Type and Recombinant Viruses Used in this Study All LCM viruses used in this study contain an identical L segment encoding for the LCMV RNA-dependent RNA polymerase (L ORF) and for the matrix protein Z. All four viruses also encode for an identical NP translation product. The three partially cDNA derived viruses (rLCMV/INDG, rLCMV/NJG, and rLCMV-ARM*) carry, however, two noncoding genetic tags in the NP ORF as indicated (*; see also Figures 4 and S6). The LCMV-GP ORF of LCMV-ARM and rLCMV-ARM* is substituted for INDG and NJG in rLCMV/INDG and rLCMV/NJG, respectively. Arrows indicate the orientation of individual ORFs. Inverted writing (NP, L) indicates that in the viral genome, the respective ORFs are encoded in antisense polarity.

Lymphocytic choriomeningitis virus (LCMV) represents the prototype member of the arenavirus family. The genome of these enveloped negative-strand RNA viruses [1] consists of a small (S) and a large (L) segment, each of them expressing two viral genes in an ambisense coding strategy (Figure 1). The S segment codes for the viral nucleoprotein (NP, approximately 63 kDa) and the glycoprotein (GP). The GP, encoded in genome polarity, is synthesized as a precursor protein GP-C (75 kDa) and posttranslational processing generates GP1/GP2 complexes forming club-shaped projections on mature infectious virions. NP, encoded in antigenome polarity, is the most abundant viral protein and encapsidates viral genomes and antigenomic replicative intermediates. The L gene segment codes for a 200-kDa L protein with characteristic hallmarks of all RNA dependent RNA polymerases and for the small 11-kDa RING finger protein Z that functions as the viral matrix protein [2].

Arenaviruses are widespread in various rodent species populating the entire globe and several of them can cause hemorrhagic fevers when accidentally transmitted to humans [3]. In addition, arenaviruses causing hemorrhagic fever have recently obtained much attention due to their potential use in warfare and bioterrorism [4, 5]. Among them are Lassa fever virus (LFV), found in West Africa, and the South American viruses Junin, Machupo, and Guanarito that cause Argentine, Bolivian, and Venezuelan hemorrhagic fever, respectively. LFV, with 100,000 to 300,000 estimated human infections and several thousand deaths, annually affects by far the largest number of humans [3]. After its first description in the late 1960s, LF was thought of as a rare disease with high case-fatality rate that was transmitted by contact in hospital epidemics. This picture has changed dramatically since it has been found that in rural areas of West Africa, LFV accounted for approximately 10% of febrile illnesses and that seroprevalence reached locally up to 50% [3]. Moreover, human-to-human transmission in a more recent nosocomial outbreak could largely be accredited to poor medical practice including needle sharing [6]. Together with the failure of LFV to spread outside the West African distribution of its rodent reservoir *Mastomys,* this indicated that the virus cannot efficiently sustain itself by human-to-human transmission [3]. Increasing numbers of imported cases due to traveling, however, are reported from around the globe [7, 8].

Ribavirin, a nucleoside analog, represents the only currently available therapy for clinical application in LF but it appears to be of limited efficacy [9]. Hence, protective vaccines are needed, but the viral immunobiology has rendered their development a difficult undertaking. Immunization with inactivated virus has failed to induce neutralizing antibodies (nAbs) or to protect against LFV in animal models [10], and convalescent serum transfer rarely increased the survival rate of LFV-infected humans [9, 11]. Accordingly, life-long protection against LFV in previously infected human subjects is commonly accredited to cell-mediated immunity [12, 13] and candidate vaccines should therefore aim at inducing long-lived T cell memory.

A variety of vector systems have been tested as potential vaccines for arenaviruses, among them DNA immunization and recombinant salmonella [14, 15]. Engineered vaccinia viruses expressing either the GP or NP of LFV have been shown to confer guinea pigs and nonhuman primates with cell-mediated immunity against lethal LFV challenge [16]. Unfortunately, the safety profile of vaccinia virus and the high HIV prevalence rate in West Africa preclude the application of this vaccine where it would be most needed [13]. The protective capacity of alphavirus-based vectors has been demonstrated in guinea pigs [17], and very recently it was shown that a recombinant vesicular stomatitis virus (VSV) expressing LFV-GP protected nonhuman primates against LF [18]. In the latter two studies, challenge infections were invariably carried out 28 d after immunization. Thus the long-term protective capacity of these vectors may require additional testing, particularly for VSV that has served as a prototype model infection eliciting only short-lived T cell−mediated protection [19]. In case of a bioterrorist attack or for health care workers and military personnel transiently deployed to endemic areas, the longevity of protection may be of less importance. For the population of West Africa, however, this will be a key criterion because of the local cumulative lifetime risk of contracting LFV ([3], see also above).

As an alternative to recombinant vector systems, heterologous Old World arenaviruses would likely represent very efficient LFV vaccines. Mopeia virus (MV) and LCMV, and recently also an LFV/MV reassortant, have been shown to protect against LF in nonhuman primates [16, 20, 21] and guinea pigs, respectively [22]. In the latter model, adoptive transfer experiments could formally show that cross-protection was cell mediated. Cross-reactivity between LCMV and LFV has also been demonstrated at the level of T cell clones and single cytotoxic T cell (CTL) epitopes [23, 24]. Moreover, the strategy of vaccinating with a closely related but less pathogenic virus (heterologous immunity) has a famous precedent in the eradication of smallpox by using vaccinia virus. Yet the safety profile of different LCMV and MV strains for humans remains unclear and untested. We therefore considered the possibility of using genetically engineered LCM viruses with a molecularly defined basis of attenuation as long-sought live-attenuated arenavirus vaccines [5, 12, 25, 26].

The arenavirus GP mediates receptor binding and cell entry, but additional roles in the viral life cycle including the efficient release of particles have recently been described (see Discussion and [2, 27−30]). GP1 represents the only target for virus neutralizing antibodies [1, 31], and we have documented a critical role of the LCMV-GP in viral evasion from efficient nAb-mediated control [31]. Similar to LCMV in mice, human LFV infection elicits commonly only vastly delayed and relatively poorly nAb responses [11, 32]. Accordingly, only the most potent convalescent sera confer passive protection against virus challenge, and prevention of lethal disease seems relatively virus isolate specific [11, 32]. Thus, cell-mediated immunity is considered the mechanism of choice for vaccine candidates [12]. α-Dystroglycan is the only characterized cellular arenavirus receptor, and the ability of certain LCMV isolates to cause overwhelming systemic infection in mice has been correlated with their high affinity binding to α-dystroglycan [33]. Interestingly, also the GP of LFV exhibits high affinity for α-dystroglycan, lending support to speculations that GP-related properties could contribute to the viral phenotype [33, 34]. Studies in guinea pigs and also in monkeys have shown that determinants on the L genome segment are primarily responsible for the viral ability to cause hemorrhagic fever and that determinants on the S segment of virulent viruses (e.g., LFV) were not sufficient for disease [21, 35]. While addressing the contribution of GP to strain-specific differences in pathogenesis, a potential need for features that are common to all arenaviral GPs had not been addressed in these experiments. Thus, we hypothesized that the reverse genetic exchange of the LCMV-GP for a foreign surface determinant may attenuate the virus and thereby may offer a general strategy for the directed engineering of live-attenuated arenavirus vaccines.

We had previously reported on a recombinant LCM virus (rLCMV/INDG) expressing the surface GP of vesicular stomatitis virus serotype Indiana (INDG) instead of its own GP (schematically depicted in Figure 1 [31, 36]). rLCMV/INDG exhibits attenuated growth in tissue culture ([31, 36], see also Figure 5A), and unlike for wild-type LCMV (LCMVwt), it elicits a rapid and protective neutralizing antibody response [31]. In the present report, we show that rLCMV/INDG has lost its pathogenic potential in mice but has retained the property of LCMVwt to induce long-lived protective CTL memory. Using newly developed techniques, we engineered two additional recombinant LCM viruses to formally accredit the attenuated phenotype of rLCMV/INDG to GP exchange and to reproduce our findings with a second attenuated virus. Our observations provide a strong rationale for testing LCMV or other arenaviruses with foreign GPs as promising live-attenuated vaccine candidates in nonhuman primate models of arenaviral diseases.

## Results

### Attenuation of rLCMV/INDG Prevents Lethal Disease in Infected Mice

**Figure 2:**
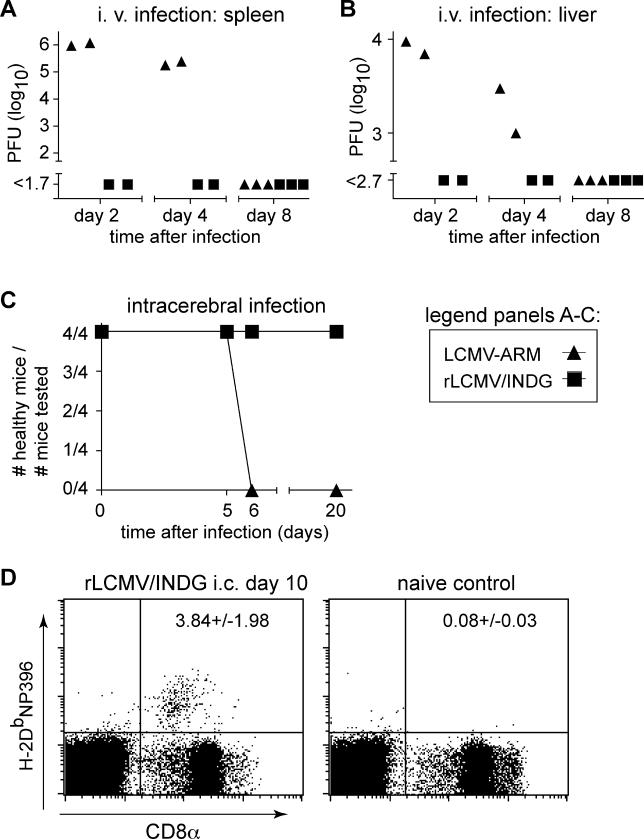
Attenuation of rLCMV/INDG Prevents Lethal Disease in Infected Mice (A, B) C57BL/6 mice were infected i.v. with 2 × 10^4^ PFU of either rLCMV/INDG or LCMV-ARM. At the indicated time points after inoculation, groups of two or three mice were killed and viral titers in spleen (A) and liver (B) were determined by immunofocus assay. Symbols represent individual mice. (C) C57BL/6 mice (four per group) were infected with 3 × 10^3^ PFU rLCMV/INDG or LCMV-ARM i.c. At the time points indicated, they were monitored for clinical signs of choriomeningitis. Animals with terminal disease were killed according to the Swiss law for animal protection. (D) Mice were infected with 3 × 10^3^ PFU rLCMV/INDG i.c. Ten days later, NP396-specific CD8^+^ T cells in peripheral blood were enumerated using MHC class I tetramers. Numbers in the upper right quadrants indicate the percentage of NP396-specific CD8^+^ T cells within the total CD8^+^ T cell population. Each panel (A–D) shows the results from one representative experiment of two similar ones.

Attenuated growth of rLCMV/INDG in cell culture [31, 36] prompted us to study its fitness and pathogenicity in infected mice. We infected C57BL/6 mice intravenously (i.v.) with 2 × 10^4^ PFU of the LCMVwt strain Armstrong (LCMV-ARM) or with the same dose of rLCMV/INDG. Groups of mice were killed 2, 4, and 8 d later to determine virus titers in spleen, liver, kidney, brain, and blood (Figure 2A and 2B and unpublished data). Following its typical kinetics [33], LCMV-ARM replicated to initially high titers in spleen and liver but was cleared to below detection limit within 6 to 8 d. In contrast, rLCMV/INDG infectivity was undetectable in all organs and at all time points tested. We therefore set out to study the pathogenic potential of rLCMV/INDG. In mice, all known LCMVwt strains including LCMV-ARM typically cause lethal lymphocytic choriomeningitis (LCM) upon intracerebral (i.c.) inoculation. This immunopathologic disease is mediated by virus-specific cytotoxic T cells, whereas the viral infection per se is noncytolytic. Severe or fatal LCM is also a frequent complication in accidental infection of human adults [37]. Little is known about the capacity of different LCMVwt isolates to cause central nervous system disease in humans, but this potential side effect of live-attenuated vaccines should be excluded as far as possible. We therefore assessed the capacity of rLCMV/INDG to elicit lethal LCM after i.c. inoculation of C57BL/6 mice (Figure 2C, Table 1). Surprisingly, rLCMV/INDG-infected mice remained clinically healthy during the entire period of observation, while LCMV-ARM control infected mice exhibited signs of terminal LCM within 6 d of infection. Notably, mice that are tolerant to LCMV-GP due to transgenic expression of this protein in the thymus (DEE mice [38]) developed normal LCM after i.c. infection with LCMV-ARM (Table 1). Thus, the failure of rLCMV/INDG to cause LCM was not due to the lack of the LCMV-GP as one of the main targets of CTL-mediated immunopathology. This indicated that GP exchange had largely abrogated the virus' pathogenic potential in mice.

**Table 1:**
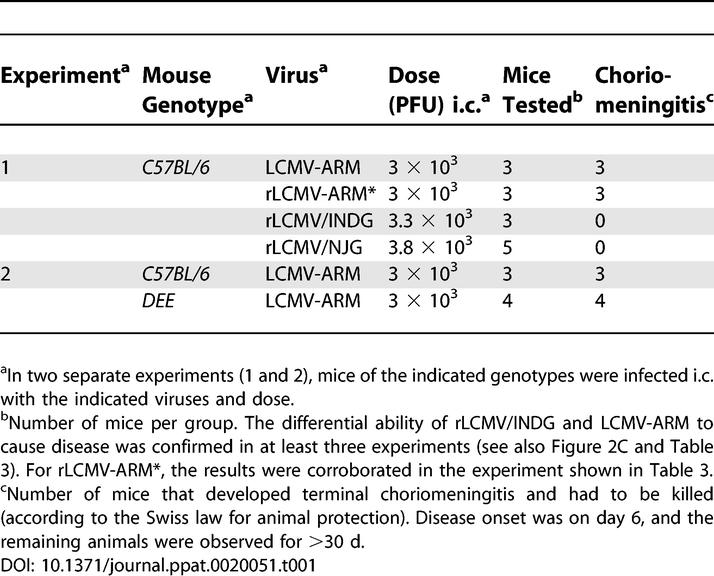
The Viral GP Determines Pathogenicity of Engineered LCM Viruses in i.c. Infected Mice

**Table 2:**
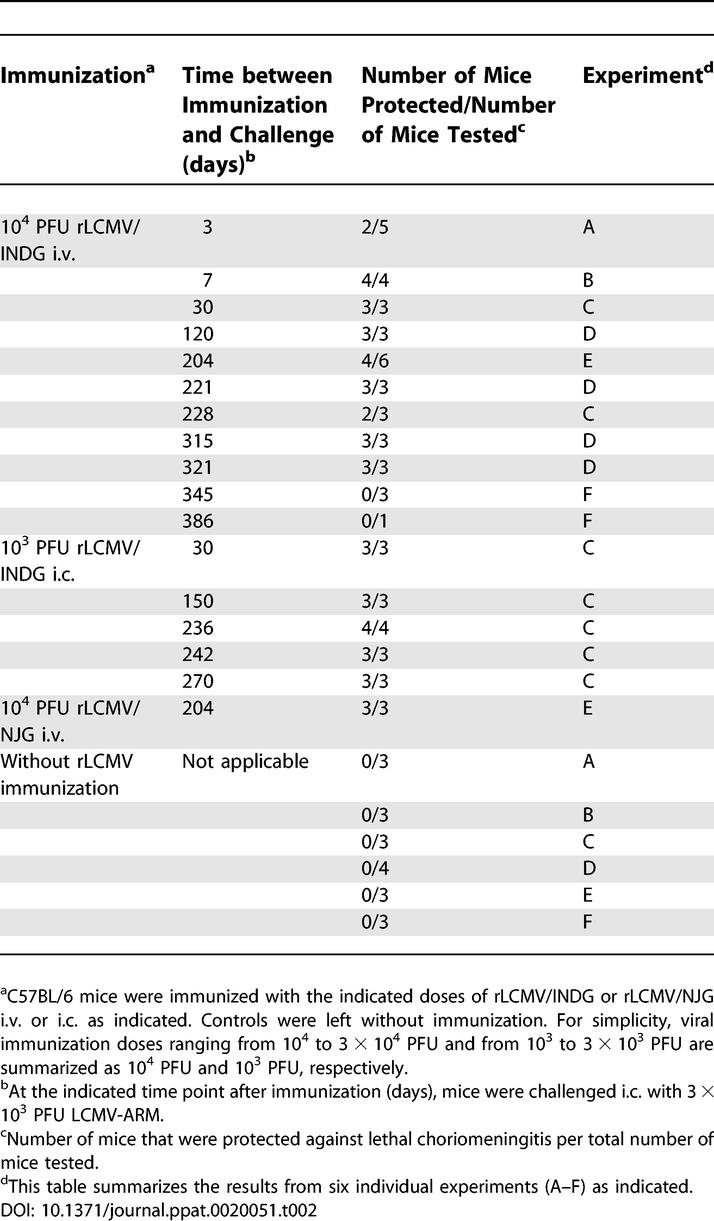
Recombinant LCM Viruses Induce Rapid and Long-Lasting Protection against Lethal Choriomeningitis

**Table 3:**
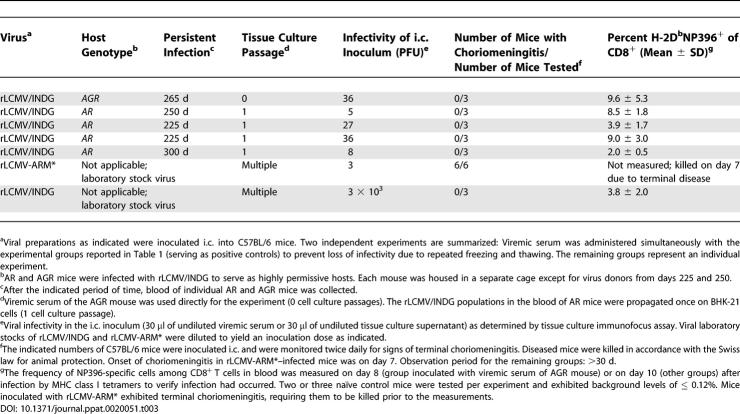
No Detectable Gain in rLCMV/INDG Pathogenicity after Propagation in Immunodeficient Hosts

### rLCMV/INDG Immunization Elicits Rapid and Long-Lived Cell-Mediated Immunity against Lethal Challenge with Wild-Type Virus

Infection with LCMVwt represents a paradigm for induction of life-long protective T cell memory [19], and we had previously found that i.v. rLCMV/INDG infection evoked not only a rapid and potent neutralizing antibody (nAb) response but also high peak frequencies of viral epitope−specific CD8^+^ T cells [31]. Here, we made analogous observations after i.c. infection with rLCMV/INDG (Figure 2D). This was not surprising considering that most of the i.c. inoculum is rapidly drained to the systemic circulation [39]. To test whether GP recombinant arenaviruses could be used as live-attenuated vaccines, we determined the protective capacity and longevity of T cell memory in rLCMV/INDG-immune C57BL/6 mice. Lethal i.c. challenge with LCMV-ARM was performed at various time points (Table 2). This setting ruled out any contribution of serum antibodies to challenge virus control because LCMV-ARM and rLCMV/INDG are exclusively neutralized by either LCMV-GP1− or INDG-specific antibodies, respectively ([31]; see also Table S1) and therefore represent distinct serotypes. In three independent experiments, a total of 21 of 24 animals immunized with 10^4^ PFU of rLCMV/INDG i.v. and all 16 mice immunized with 10^3^ PFU of rLCMV/INDG i.c. survived a lethal i.c. challenge with LCMV-ARM at time points ranging from 30 to 321 d after immunization (Table 2). This protective effect of rLCMV/INDG immunization correlated with the rapid expansion of virus-specific memory CTL and with efficient clearance of LCMV-ARM from the central nervous system before infection became too widespread (for details, see Protocol S1 and Figure S1). A lack of protection in a challenge experiment carried out at days 345 and 386 suggested, however, that protection might not last infinitely.

In contrast to the needs in endemic areas (longevity of protection), a vaccine for health care workers or military personnel deployed to such places should primarily provide rapid protection. The same criterion would also apply in case of a bioterrorist attack. Thus, it was important to find that rLCMV/INDG immunization was protective even when administered no earlier than 7 d prior to i.c. challenge with LCMV-ARM and that protection was even seen in two of five animals challenged on day 3 after immunization (Table 2).

### rLCMV/INDG Immunization Confers Rapid and Long-Lived Cell-Mediated Protection against Overwhelming Systemic Infection and Liver Disease

**Figure 3:**
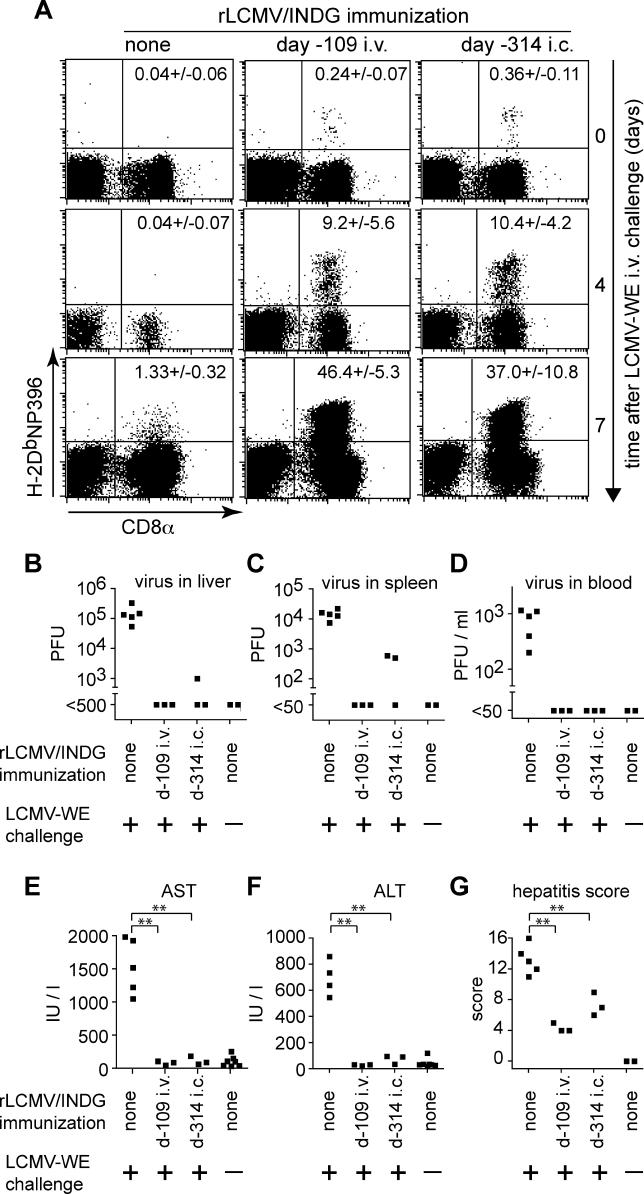
Efficient Control of High-Dose LCMV-WE Infection and Prevention of Liver Disease by rLCMV/INDG-Induced Memory CTL (A) C57BL/6 mice were infected with 2 × 10^4^ PFU rLCMV/INDG i.v. or with 3 × 10^3^ PFU rLCMV/INDG i.c. on day −109 and on day −314, respectively. On day 0, the immunized mice and a group of naïve control mice (“none”) were challenged with 2 × 10^5^ PFU LCMV-WE i.v. The frequency of NP396-specific memory CD8^+^ T cells in peripheral blood was assessed prior to challenge (day 0) and on day 4 and day 7 after challenge using MHC class I tetramers. The numbers in the upper right quadrants indicate the frequency of NP396-specific CD8^+^ T cells within the total CD8^+^ T cell population in peripheral blood and represent the mean ± SD of three or four mice per group and time point. (B–D) All mice were killed 7 d after challenge, and viral titers in liver, spleen, and blood were determined by immunofocus assay. (E, F) On day 7, serum AST and ALT levels were determined. (G) Liver sections were scored for hepatitis. The symbols in B–G represent individual mice. Statistical analysis was carried out for E–G. Significant differences between groups are indicated for **P < 0.01. All findings described were reproduced in the experiment shown in Figure S5.

To confer protection against arenaviral hemorrhagic fevers, vaccine-induced cell-mediated immunity should prevent overwhelming viremia, the primary predictor for the outcome of human LFV infection [9]. Moreover, efficient virus control should avoid the disturbance of clinical parameters predictive for an unfavorable outcome, among them elevated serum aspartate aminotransferase (AST) activity, which is used to monitor human LF [9]. In mice, high-dose infection with the viscerotropic WE strain of LCMV results in prolonged viremia, severe AST elevation, and occasionally a fatal outcome [40, 41]. We therefore tested whether immunization with rLCMV/INDG could protect mice against high-dose LCMV-WE challenge. Considering the similarly efficient immune protection conferred by i.c. or i.v. infection with rLCMV/INDG (Table 2), mice that had originally been inoculated via either route were used likewise for the subsequent sets of experiments. The peripheral CD8^+^ T cell pool of mice immunized 109 and 314 d previously by i.v. or i.c. inoculation of rLCMV/INDG, respectively, contained approximately 0.2% to 0.5% NP396-specific memory CTL (Figure 3A). Upon high-dose (2 × 10^5^ PFU) LCMV-WE i.v. infection (sharing the NP396 epitope with rLCMV/INDG), these cells expanded to approximately 10% by day 4 and to approximately 30% to 50% by day 7 after challenge. In naïve control animals, only approximately 1% to 2% NP396-specific CD8^+^ T cells were detected no earlier than 7 d after challenge. At this time point, mice without immunization exhibited high virus load in blood, spleen, and liver, whereas the virus had been cleared to below detectable levels in 109-d rLCMV/INDG i.v. immune animals (Figure 3B–3D). Even in mice that had been infected with rLCMV/INDG i.c. 314 d prior to challenge, virus load was either considerably reduced or undetectable. This was reflected in serum AST and alanine aminotransferase (ALT) levels of immune mice that remained in the normal range, whereas the corresponding values of nonimmune control mice reflected serious disease (Figure 3E and 3F). Histopathologic analysis of the liver 7 d after challenge infection documented clearance of viral antigen at the cellular level and revealed considerably milder mononuclear infiltrations in the liver of rLCMV/INDG-immune mice (Figure S2). The reduction in hepatocellular damage was also reflected in a lower histopathological hepatitis score (Figure 3G [42]). In the i.c. challenge model with LCMV-ARM (Table 2), we had found that rLCMV/INDG-induced protection was rapidly established. Analogous observations were made when mice were challenged with a high dose of LCMV-WE i.v. either 3 or 6 d after immunization (see Protocol S1 and Figure S3). Taken together, these experiments showed that rLCMV/INDG immunization provided rapid and long-lived cell-mediated immunity against overwhelming systemic infection and liver disease elicited by a viscerotropic LCMV isolate.

### A Second GP Exchange Virus Reproduces the Phenotype of rLCMV/INDG

**Figure 4:**
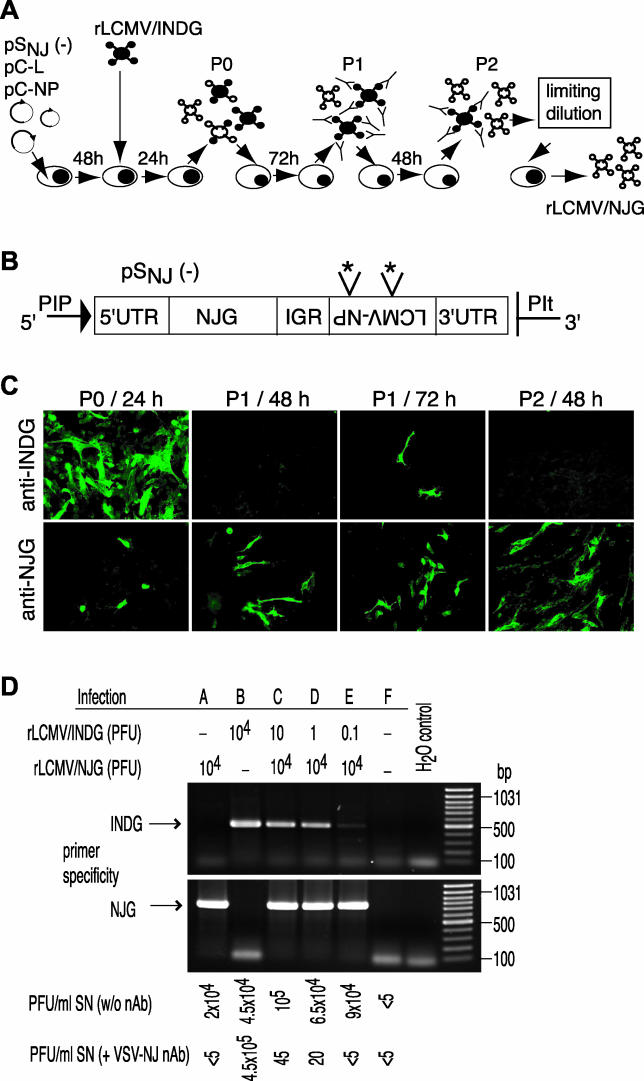
Generation and Characterization of rLCMV/NJG (A) Schematic describing the protocol for generation of rLCMV/NJG. (B) pS_NJ_(−) was designed analogous to pS_IND_ (also referred to as pSr(−) [36]). It expresses in genomic (−) polarity a recombinant LCMV S segment where the LCMV-GP ORF was substituted for the *NJG* gene. Transcription is driven by the murine polymerase I promotor (PIP) and is terminated upstream of the murine polymerase I terminator (PIT). UTR, untranslated region; IGR, intergenic region; *noncoding single nucleotide tags. (C) BHK-21 cells on duplicate coverslips were infected for 24 h with supernatants collected from the experiment outlined in A. Staining with INDG- or NJG-specific mAbs as indicated provided a rough estimate of the relative proportions of rLCMV/INDG and rLCMV/NJG contained in the supernatants tested. (D) BHK-21 cells (10^6^ per M6 tissue culture well) were infected with different doses of rLCMV/INDG or of rLCMV/NJG or with both viruses in different combinations as indicated in the chart. At 48 h later, the cells as well as the supernatant were collected. Total cellular RNA was extracted and was processed for detection of INDG and NJG RNA by RT-PCR. The PCR products were separated by agarose gel electrophoresis and were visualized by ethidium bromide staining. Specific amplification products of the expected size are indicated with *arrows*. Viral infectivity in the supernatant (SN) was measured by immunofocus assay [PFU/ml SN (w/o nAb) listed in the chart under the gel picture]. In addition, an aliquot of supernatant was incubated with VSV-NJ neutralizing mAb H6B9D5 prior to testing the remaining infectivity [see PFU/ml SN (+VSV-NJ nAb)] to discriminate rLCMV/INDG from rLCMV/NJG infectivity.

The high degree of attenuation of rLCMV/INDG combined with its protective capacity suggested that GP exchange represented a promising general strategy to attenuate arenaviruses for use as live vaccines. It remained, however, to be formally demonstrated that GP exchange was indeed the main reason for the attenuated phenotype of rLCMV/INDG. Randomly accumulated mutations in the virus' RNA genome could also have accounted for a loss of fitness. Moreover, the partial cDNA origin of rLCMV/INDG per se could have caused some attenuation [43]. This prompted us to determine whether the phenotype of rLCMV/INDG was reproducible with another GP exchange virus. For this, we generated a recombinant LCMV (rLCMV/NJG, Figure 1) expressing the GP of the New Jersey (NJ) serotype of VSV instead of INDG or LCMV-GP. rLCMV/INDG had so far represented the only genetically engineered recombinant arenavirus, and the protocol we had used for its generation had been extremely labor intensive [36]. For the recovery of rLCMV/NJG, we therefore developed a new experimental protocol that was, like the original protocol, based on the reassortment of viral genome segments (schematically depicted in Figure 4A). We transfected BHK-21 cells with a polymerase I (pol-I)−driven vector [pS_NJ_(−), Figure 4B] for intracellular expression of an NJG recombinant LCMV S segment RNA in combination with polymerase II (pol-II)-driven plasmids for coexpression of the minimal viral transacting factors NP and L (pC-NP, pC-L [2, 36]). Forty-eight hours later when recombinant S segment ribonucleoproteins had formed intracellularly [36], the transfected cells were infected with rLCMV/INDG helper virus, providing an L genome segment for reassortment. The appearance of rLCMV/NJG reassortant virus in the supernatant and the subsequent selection process were monitored by immunofluorescence. For this, fresh BHK-21 cells were incubated with culture supernatant sampled at different time points, and the rLCMV/INDG- or rLCMV/NJG-specific infectivity contained therein was detected 24 h later in a semiquantitative manner by INDG- or NJG-specific immunofluorescence staining (Figure 4C). Supernatant collected 24 h after helper virus infection contained already a low but detectable fraction of rLCMV/NJG reassortant virus (Figure 4C, P0/24h). To select against the dominant population of rLCMV/INDG helper virus, the mixed virus population was propagated for two consecutive passages (P1 and P2 in Figure 4A and 4C) on fresh BHK-21 cells in the presence of INDG-neutralizing monoclonal antibody (VI-7 [31]). This procedure resulted in a substantial enrichment of rLCMV/NJG and simultaneously in the loss of rLCMV/INDG to below detectable levels by immunofluorescence. The so-obtained population of rLCMV/NJG was subject to a single round of limiting dilution, and the absence of rLCMV/INDG helper virus in rLCMV/NJG (<1 PFU in 10^4^ PFU of rLCMV/NJG) was verified by RT-PCR (Figure 4D).

**Figure 5:**
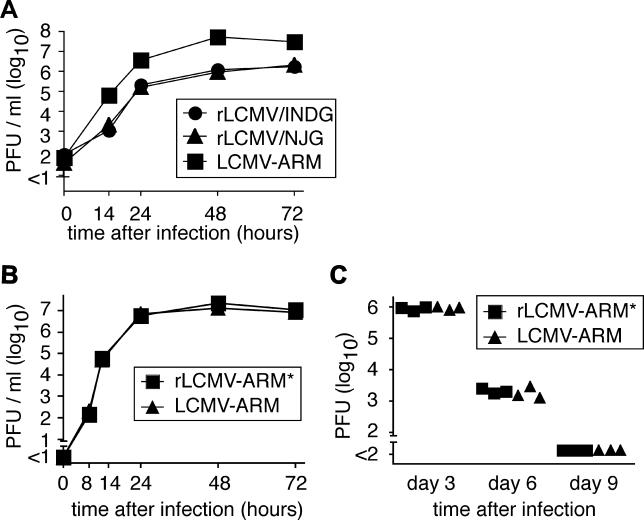
Attenuated Propagation of rLCMV/NJG but Not of rLCMV-ARM* (A) BHK-21 cells (10^6^ per M6 well) were infected with the indicated viruses at a multiplicity of infection of 0.01, and infectious virus in the supernatant was measured at the indicated time points. Symbols indicate the mean of three tissue culture wells (SD bars project into the symbol size). (B) BHK-21 cells in M6 tissue culture wells were infected with LCMV-ARM or rLCMV-ARM* at multiplicity of infection of 0.01, and infectious virus in the supernatant was measured at the indicated time points. The symbols represent values from a single cell culture well. One representative experiment of two is shown. (C) C57BL/6 mice infected i.v. with 10^5^ PFU LCMV-ARM or rLCMV-ARM* were killed at the indicated time points, and virus titers in spleen were measured. Symbols represent individual mice. Analogous results were obtained from liver tissue (not shown). One representative experiment of two is shown.

Characterization of rLCMV/NJG in cell culture (Table S1) showed that this virus was neutralized by VSV-NJ−specific, but not by VSV-IND− or LCMV-ARM−specific, monoclonal antibodies (mAbs), demonstrating that receptor binding and cell fusion of rLCMV/NJG were mediated by the foreign NJG protein as expected. Moreover, we observed that rLCMV/NJG infection elicited a very rapid and potent serotype-specific nAb response (Protocol S1 and Figure S4) as previously reported for rLCMV/INDG [31]. Most important, however, rLCMV/NJG and rLCMV/INDG exhibited similarly attenuated growth in cell culture when compared to LCMV-ARM (Figure 5A). In accordance with this, rLCMV/NJG failed to induce clinically apparent disease in i.c. infected mice as previously observed with rLCMV/INDG (Table 1). In further analogy, a single i.v. immunization with rLCMV/NJG protected against lethal i.c. challenge with LCMV-ARM (Table 2) or conferred long-lived CTL memory and resistance against high-dose i.v. challenge with LCMV-WE (Figure S5, compare to Figure 3).

### Wild-Type Pathogenicity of an Engineered LCM Virus Expressing LCMV-GP

Next, we addressed the possibility that the attenuation of rLCMV/INDG and of rLCMV/NJG was due to their partial origin from cDNA [43] or that it was a result of randomly accumulated mutations in the large genome segment. We therefore substituted the recombinant S segment in rLCMV/INDG for a cDNA-derived and genetically tagged S segment encoding for wild-type LCMV-GP (Figures 1A and S6). The new virus, named rLCMV-ARM*, was recovered by the same strategy as described for rLCMV/NJG (Figure 4A). Additional technical details and a genetic characterization of rLCMV-ARM* are provided in protocol S1 and in Figure S6. rLCMV-ARM* was neutralized by the same mAbs and hyperimmune sera as wild-type LCMV-ARM (Table S1) and, even more important, propagation of rLCMV-ARM* in cell culture was indistinguishable from LCMV-ARM (Figure 5B). To determine the fitness of rLCMV-ARM* in vivo, we infected mice i.v. with 10^5^ PFU of either rLCMV-ARM* or LCMV-ARM and determined viral titers in the spleen 3, 6, and 9 d later (Figure 5C). Virus load and the kinetics of clearance were identical in both groups. In accordance with this, the CD8^+^ T cell responses elicited by the two viruses were indistinguishable (Protocol S1 and Figure S7). As the most stringent test for pathogenicity, we performed i.c. inoculation experiments. Infection of C57BL/6 mice with 3 × 10^3^ or even with only 3 PFU of rLCMV-ARM* was invariably lethal (Tables 1 and 3), demonstrating that rLCMV-ARM* behaved like wild-type LCMV-ARM. Thus, the attenuation of rLCMV/INDG and of rLCMV/NJG was due to GP exchange and could not be attributed to their partial origin from cDNA or to accumulated mutations in their L genome segment.

### No Detectable Gain in rLCMV/INDG Pathogenicity after Propagation in Immunodeficient Hosts

A major concern and a common drawback of live-attenuated viral vaccines lie in their potential for reversion [44]. In particular, viral variants of increased fitness and pathogenicity may be selected during persistent infection of immunocompromized vaccinees that continue to shed virus [45]. We therefore assessed how easily rLCMV/INDG could evolve into a virus that was pathogenic for mice. For this, we infected five either interferon type I receptor^−/−^ RAG^−/−^ double deficient mice (AR) or interferon type I receptor^−/−^ interferon type II receptor^−/−^ RAG^−/−^ triple deficient mice (AGR [46]) with rLCMV/INDG and kept them in three separate cages for 225 to 300 d (Table 3) to prevent virus transmission between mice. The combined genetic deficiencies of these animals allowed rLCMV/INDG to readily establish viremia and to replicate systemically (AB, DM, and DDP, unpublished data), which should facilitate the evolution of the viral quasi-species and the emergence of more pathogenic variants [47]. Notably, rLCMV/INDG viremia in these mice did not cause clinically apparent disease, confirming our earlier observation that this virus is noncytolytic in vivo [36], which may be of importance with regard to vaccine safety. To test for a clear increase in viral pathogenicity after 225 to 300 d of in vivo replication, we inoculated C57BL/6 mice i.c. with viremic serum or with undiluted supernatant from a single cell culture passage of blood-derived virus (Table 3). Mouse-passaged rLCMV/INDG preparations contained no more than approximately 10^2^ to 10^3^ PFU/ml infectious virus due to low level viremia even in AGR mice and relatively poor in vitro amplification of ex vivo isolated rLCMV/INDG (unpublished data). Accordingly, the i.c. inoculum that could be administered to C57BL/6 indicator mice was relatively low (<50 PFU in 30 μl, see Table 3). Still, all viral preparations tested elicited high frequencies of epitope-specific CD8^+^ T cells indicative for a productive infection, but the infected animals remained clinically healthy. This failure of mouse-passaged rLCMV/INDG to induce disease could unlikely be attributed solely to dose limitations but suggested stable attenuation. Even an i.c. inoculum of a single PFU of LCMV-ARM is lethal in mice [41], and analogous findings were made here with an i.c. inoculum of only 3 PFU of rLCMV-ARM* (Table 3). Furthermore, the finding that only low amounts of virus were recovered from the serum of AR and AGR mice provided per se additional support for this notion. Nevertheless, additional testing will be needed to confirm and extend these findings, and a certain increase in fitness concomitant with prolonged or even repeated in vivo passage of rLCMV/INDG seems likely and cannot be excluded at present [47]. Similarly, limitations in the volume and hence in the amount of infectivity that could be administered i.c. have so far precluded the formal assessment of a 50% lethal dose (LD_50_) for rLCMV/INDG, rLCMV/NJG, or any of the ex vivo isolated virus populations shown in Table 3. Considering, however, that even the administration of low doses (1 to 10 PFU) of rLCMV/INDG elicited considerable frequencies of antiviral CTL, whereas i.c. infections with up to 10^4^ PFU of rLCMV/INDG failed to elicit clinical disease (Figure 2C and Tables 1 and 3, and unpublished data), the safety margin for such a vaccine is likely to be broad, i.e., ≥100-fold the dose needed for vaccination. Even arenaviruses with foreign GPs are, however, unlikely to be considered for application in pregnant women or in newborns, owing to the teratogenic potential of their parent viruses. Also, we had previously reported that rLCMV/INDG persists in neonates, and it was therefore of concern that the virus might also persist in the central nervous system when administered in adult life. rLCMV/INDG was detectable in the meninges by immunohistochemistry around 4 d after i.c. inoculation of adult mice although at lower levels than LCMVwt. Yet, the virus was cleared to below detection levels of our RT-PCR assay within 2 wk, whereas it persisted in neonatally infected animals (Figure S8 and unpublished data). Also, upon i.v. infection, viral RNA in the serum was detectable by RT-PCR on only day 4 but not on day 8 (unpublished data), alleviating potential concerns regarding the virus' ability to establish a persistent infection in adult immunocompetent individuals. It is clear, however, that arenaviruses with foreign GPs need to be investigated in much more detail, specifically also in nonhuman primate models, before projections on their use in the human population can be made.

## Discussion

The directed introduction of mutations in viral genes often results in a complete loss of function [48], abrogating the virus' viability and thereby also its immunogenicity. Conversely, even RNA viruses carrying multiple attenuating point mutations are prone to reversion [44]. Hence, the deletion of a dispensable nonstructural gene [49] or the substitution of a structural gene for a foreign protein like in the present report may provide an advantageous new strategy. Arenaviruses have only four known genes and hence the range of potential targets is limited. As supported by recent mutagenesis studies, major changes in the minimal viral transacting factors NP or L would unlikely be tolerated [48]. Z mutants with residual budding activity have been characterized [2], and therefore the viral matrix protein may represent an attractive target for attenuating mutagenesis. Yet, the concerns related to the possibility of back mutation and phenotypic reversion would remain. In this respect, exchange of the arenavirus GP for foreign surface determinants like VSVG may be entirely different because the roles of LCMV-GP in the viral life cycle are multifaceted. Recent studies have shown that the signal peptide of LCMV-GP serves not only to target the nascent GPC polypeptide to the endoplasmic reticulum: It is apparently needed at multiple steps of GP biosynthesis and trafficking as well as for its functioning in the viral envelope [27–29]. More important, the signal peptide is efficiently packaged in viral particles and is thought to contribute to the budding process by supporting membrane curvature [27]. Highly specific functions are also located in the C-terminus of arenavirus GPs. A cluster of basic amino acid residues in the intracellular portion of LCMV-GP is supposed to mediate critical protein-protein interactions [30]. Such interplay includes with all likelihood the binding of GP to intracellular viral components and thereby either directly or indirectly with the ribonucleoprotein [50], allowing for the efficient recruitment of GP molecules into viral particles. The INDG or NJG proteins of the engineered viruses can apparently mediate receptor binding and cell fusion, whereas additional roles of LCMV-GP including those mentioned above are likely to remain without substitute. To recover these functions of LCMV-GP in the virus' life cycle, a foreign GP like INDG or NJG would need to undergo a major evolution, including de novo development of functional domains, e.g., those in the GP signal peptide and C-terminal portion but probably many more. These deficiencies may therefore provide the molecular basis for the relatively stable, attenuated phenotype observed in this study. Alternatively and not mutually exclusively, INDG- or NJG-mediated cell tropism in the infected central nervous system or the reported differences in the humoral and cellular immune response ([31], see also Figure S4) or both could account for the lack of disease in rLCMV/INDG or rLCMV/NJG i.c. infected mice. Preliminary results support, however, the notion that the cell tropism of LCMV-ARM and of rLCMV/INDG in the acutely infected central nervous system are largely identical (DM and DDP, unpublished data). It rather appears that a lower viral burden in the rLCMV/INDG-infected central nervous system shifts virus-host balance in favor of the rLCMV/INDG-infected host (similarly to LCMV-ARM infection in immune mice, see Figure S1). Although control of rLCMV/INDG and rLCMV/NJG is unaffected in B cell–deficient mice ([31] and AB and DDP, unpublished data), the rapid and strong induction of nAbs (Figure S4) is likely to contribute to the efficient elimination of these viruses. Increased susceptibility to the host's innate immune defense represents an alternative and not mutually exclusive explanation that is currently under investigation (AB, DM, and DDP, unpublished data).

A number of recombinant vector systems have been developed as potential LFV vaccines ([14–18], see introduction section). The attenuated Junin virus variant Candid #1 is, however, the only arenavirus vaccine ever in clinical use. It has successfully been administered to agricultural workers at immediate risk of contracting Junin virus, the causative agent of Argentine hemorrhagic fever [25]. Concerns about its phenotypic stability and safety [26] render it, however, inappropriate for mass vaccinations. Accordingly, strategies like recombinant vector systems and DNA vaccination may be the methods of choice to protect entire populations at only low risk or at unpredictable risk (e.g., bioterrorist attacks), because these vaccination protocols can be tuned for an optimal safety profile. In highly affected populations (see above), efficacy and safety may be balanced differently. Moreover, logistic constraints in Third World countries where LFV and other arenaviruses are endemic will not allow for regular booster immunizations. Under such conditions, not only the efficacy but also the longevity of vaccine protection becomes a primary criterion that is commonly best fulfilled by live-attenuated vaccines. Hence the development of recombinant vaccine vectors but also of live-attenuated vaccines should be pursued both in parallel and as complementary strategies for different needs in different areas of the world.

For arenavirus family members other than LCMV, genetically engineered infectious viruses have not been reported so far. The approach outlined here and in recent work [36, 51, 52] should be applicable to all other arenaviruses including the high-risk pathogens. Thereby, attenuated viruses could be tailored for the purpose of vaccination and, if needed, could be adapted upon testing in animal models including nonhuman primates. This should allow for the rationale optimization of an LFV vaccine strain to be used in endemic areas or in cases of imminent bioterrorist attacks. Based on the well-established phenomenon of heterologous cell-mediated immunity between different Old World arenaviruses ([16, 20, 22–24], see introduction section), rLCMV/INDG and rLCMV/NJG themselves should also be considered for testing as LFV vaccines. Cell-mediated immunity to the viral NP is protective in the guinea pig model and, at a challenge dose of 10^3^ PFU, protection was also observed in nonhuman primates [16]. This is a likely dose scenario for accidental exposure to persistently infected Mastomys urine (~10^3^ PFU/ml [53]). When a challenge dose of 10^4^ PFU was given to primates, LFV-GP–specific T cell immunity seemed of superior protective capacity [16]. These differences may be due to epitope dominance in the specific monkeys tested, something that is primarily related to the individual's MHC haplotype and less to the species [54]. In support of this notion, studies on human CD4^+^ T cell immunity to LFV have documented strong NP, as well as GP, reactivity, and epitope-specific responses were found to correlate with the individual's MHC haplotype [55, 56]. Moreover, immune responses to subdominant viral epitopes can also be protective [57]. Thus, arenaviruses with foreign GPs inducing potent cell-mediated immunity against NP, but not GP, and depending on the haplotype, potentially also against Z and L, should in principle have the capacity to protect. In any case, however, GP-specific immunity [23] would be beneficial, also with regard to a potential additive effect of GP-specific neutralizing antibodies [11]. As an alternative attractive possibility, rLCMV/INDG or rLCMV/NJG or both in a sequential order (see later) could therefore be used to prevent potential side effects of a subsequent immunization with wild-type arenaviruses like LCMV, MV, or reassortants that are known to confer efficient long-term protection against LF [16, 20–22]. An analogous approach has recently been put forward in a study for smallpox vaccination [58].

Similar to LCMV-WE infection of mice ([40], compare to Figures 3, S2, S3, and S5), a human LFV vaccine should limit systemic virus spread at an early stage [9, 59]. As a further analogy to LCMV infection of mice [36], LFV in humans and nonhuman primates seems primarily controlled by cellular [12, 13, 22] rather than humoral [9, 11, 12] immunity, and hence a vaccine should induce long-lived memory CTLs. We cannot exclude, however, that mechanisms of viral interference, either directly or via the activation of innate immunity [60, 61], may have contributed to the protective effects observed very early after vaccination, i.e., on day 3 (compare Table 2 and Figure S3). The mechanisms underlying the ability of rLCMV/INDG and rLCMV/NJG to elicit long-lived cell-mediated immunity have not yet been elucidated. It seems likely, though, that their noncytolytic behavior facilitates prolonged antigen presentation by infected dendritic cells, similar to the human yellow fever vaccine [62]. Still, immune protection could likely be optimized by prime-boost regimens. After immunization with an engineered virus like rLCMV/INDG, however, the induction of nAbs to the respective GP ([31], Figure S4) would likely interfere with efficient boosting. The sequential application of different serotypes like rLCMV/INDG and rLCMV/NJG should circumvent this problem, and VSV-GPs offer excellent tools for this purpose, also because the vast majority of the human population lacks preexisting humoral immunity to VSV. In rural areas where logistical constraints represent a major limitation, recombinant expression of a viral GP from an unrelated human pathogen would offer the additional advantage of simultaneously protecting not only against arenaviral disease but also against an unrelated third party virus with similar distribution (e.g., LFV and yellow fever virus in the case of West Africa [12, 13]).

Taken together, the experiments presented herein demonstrate that the reverse genetic engineering of stably attenuated live arenavirus vaccines is feasible. They show also that abrogation of arenaviral pathogenicity does not necessarily go along with a reduction of immunogenicity to below a critical threshold needed for protective immunity. The identification of the GP as an Achilles' heel of arenaviruses offers an attractive strategy, and together with the technological advances presented here and elsewhere [36, 51, 52] may provide a basis for the engineering of live-attenuated vaccine strains. We hope that this may pave the way to the development of efficient preventive measures against hemorrhagic fevers that every year affect hundreds of thousands in West Africa and become increasingly distributed over the entire globe [7, 8].

## Materials and Methods

### Mice and animal experiments.

C57BL/6 mice, interferon type I receptor^−/−^ RAG^−/−^ double deficient mice, interferon type I receptor^−/−^ interferon type II receptor^−/−^ RAG^−/−^ triple deficient mice [46], and DEE mice [38] were bred at the Institut für Labortierkunde, University of Zurich, Zurich, Switzerland, and were housed under SPF conditions for the experiments. The i.c. infected mice exhibiting signs of terminal choriomeningitis (shaky walk, trembling, convulsions) were killed according to the Swiss law for animal protection. Mice infected with LCMV-ARM or rLCMV-ARM* were monitored twice daily during the critical period. All animal experiments were carried out with authorization of the Veterinäramt of the Kanton Zürich and in accordance with the Swiss law.

### Enumeration of epitope-specific CD8^+^ T cells.

Epitope-specific CD8^+^ T cells were stained with APC- or PE-conjugated MHC class I tetramers containing the immunodominant H-2D^b^ restricted LCMV epitopes NP396–404 (NP396) or GP33–41 (GP33) as previously described [31]. Costaining was performed with anti–CD8α-FITC or anti–CD8α-PE conjugate (clone 53–6.7) and with anti–B220-PerCP conjugate obtained from BD PharMingen (San Diego, California, United States). The cells were measured on a FACScalibur (Becton Dickinson, San Diego, California, United States), and the frequency of tetramer^+^CD8^+^ cells was calculated as percentage of the CD8^+^B220^–^ lymphocyte population. All FACS plots shown are gated on B220^−^ lymphocytes.

### Viruses and virus titration.

LCMV Armstrong ARM5.3b (LCMV-ARM) is a triple plaque purified isolate of ARM CA 1371 obtained originally from M. J. Buchmeier (The Scripps Research Institute, La Jolla, California, United States). LCMV-WE was originally obtained from F. Lehmann-Grube (Heinrich-Pette Institut, Hamburg, Germany). Generation of rLCMV/INDG has been described [36]. VSV serotype Indiana (VSV-IND) and VSV-NJ were originally obtained from D. Kolakofsky (University of Geneva, Geneva, Switzerland). LCMV-WE was propagated on L-929 cells. All other viruses were grown on BHK-21 cells. LCMV and recombinants were titrated by immunofocus assay on MC57 cells as previously described [31].

### Statistical analysis.

For the assessment of between-group differences in experiments with more than two groups, we performed one-way ANOVA followed by multiple *t-*test (with Bonferroni adjustment for multiple comparison) if the *F*-test of ANOVA indicated statistically significant differences. Student's *t*-test was used for experiments with only two groups. The analysis was carried out using GraphPad Prism software (Version 4.0b; GraphPad Software, San Diego, California). *P*-values <0.05 were considered statistically significant (*), *P*-values <0.01 were considered highly significant (**), and they are indicated in the figures accordingly (* or **).

### Histopathological scoring.

Animals were lethally anesthetized with pentobarbital and were transcardially perfused with saline followed by 4% para-formaldehyde (pH 7.4). The liver was dissected and postfixed overnight in fresh fixative followed by paraffin embedding. Histological evaluation was performed on 3-μm-thick sections stained with hematoxylin-eosin (H-E). Hepatitis was scored from 0 (healthy naïve controls) to a cumulative maximal score of 18 points according to established criteria [42]. In brief, this necroinflammatory score considers portal, periportal, and intra-acinar inflammatory cell infiltration as well as various forms of liver cell damage and necrosis: spotty (focal) lytic necrosis and apoptosis with liver cell drop-out, piecemeal necrosis (interface hepatitis), simple confluent necrosis (death of groups of adjacent hepatocytes without clear zonal location or bridging), zonal confluent necrosis (zone 3), bridging necrosis linking vascular structures, and panacinar or multiacinar necrosis. For each animal, at least ten high-power fields were analyzed.

## Supporting Information

Figure S1 Rapid Elimination of Intracerebral LCMV-ARM Infection by rLCMV/INDG-Induced Memory CTL (A) C57BL/6 mice were infected with 3 × 10^3^ PFU rLCMV/INDG i.c. on day −264. On day 0, the immunized mice and a group of naïve control mice (“none”) were challenged with 3 × 10^3^ PFU LCMV-ARM i.c. The frequency of NP396-specific memory CD8^+^ T cells in peripheral blood was assessed prior to challenge (day 0) and 4 d after challenge using MHC class I tetramers. The numbers in the upper right quadrants indicate the frequency of NP396-specific CD8^+^ T cells within the total CD8^+^ T cell population in peripheral blood and represent the mean ± SD of three or four mice per group and time point.
(B) All mice were killed 5 d after challenge, and viral titers in brain and spleen were determined by immunofocus assay. Symbols indicate individual mice.
(405 KB EPS)

Figure S2 Efficient Clearance of LCMV-WE and Blunted Liver Disease in rLCMV/INDG-Immune Mice C57BL/6 mice were infected with 2 × 10^4^ PFU rLCMV/INDG i.v. or with 3 × 10^3^ PFU rLCMV/INDG i.c. on day −109 and day −314, respectively. On day 0, the immunized mice and a group of previously naïve control mice (“no immunization”) were challenged with 2 × 10^5^ PFU LCMV-WE i.v. (same animals as in Figure 3). Seven days later, the mice were killed, and either liver tissue was stained with H-E (left column) or immunofluorescent costaining was performed (right column) to simultaneously detect LCMV antigen (green), CD3^+^ T cells (red), and cell nuclei (DAPI, blue). Liver tissue samples of three mice per group were analyzed, except for the group without challenge consisting of two naïve mice (negative control). The pictures shown were taken from areas with a representative degree of inflammation and with representative amounts of viral antigen. The hepatitis score for the same samples is shown in Figure 4G. An analogous histopathological picture was also seen in tissue sections collected in the experiment described in Figure S5 (not shown).
(11 MB EPS)

Figure S3 rLCMV/INDG-Induced Immune Protection Is Rapidly Established In two separate experiments shown in A–E and F–J, respectively, C57BL/6 mice were either left untreated or immunized with 2 × 10^4^ PFU rLCMV/INDG i.v. prior to challenge with LCMV-WE (2 × 10^5^ PFU i.v.). The immunization was performed either 6 d (A–E) or 3 d (F–J) prior to challenge. Eight days after challenge, the mice were killed and virus titers were determined in blood (A and F), spleen (B and G), and liver (C and H). In addition, AST (D and I) and ALT (E and J) activity was determined in the serum. The symbols represent individual mice. For each experiment (A–E and F–J), one representative of two is shown. Statistical analysis was carried out for D, E, I, and J, and significant differences between groups are indicated as *P < 0.05 or **P < 0.01.
(402 KB EPS)

Figure S4 Rapid and Potent nAb Response to rLCMV/NJG Infection C57BL/6 mice were infected i.v. with 2 × 10^4^ PFU of rLCMV/INDG, rLCMV/NJG, VSV-IND, or VSV-NJ. Serum was collected at the indicated time points and was prediluted 40-fold for testing in plaque reduction assays. Neutralizing IgM (A and C) and IgG (B and D) against VSV-NJ (A and B) and against VSV-IND (C and D), respectively, were measured as outlined in the chart. Symbols represent the mean ± SD of three mice.
(389 KB EPS)

Figure S5 Efficient CTL Recall Response, Control of High-Dose LCMV-WE Infection, and Prevention of Liver Disease in rLCMV/INDG- or rLCMV/NJG-Immune Mice (A) C57BL/6 mice were infected with 3 × 10^3^ PFU rLCMV/INDG or rLCMV/NJG i.c. on day −156. On day 0, the immunized mice and a group of naïve control mice (“none”) were challenged with 2 × 10^5^ PFU LCMV-WE i.v. The frequency of NP396-specific memory CD8^+^ T cells in peripheral blood was assessed prior to challenge (day 0), on day 4, and on day 7 after challenge using MHC class I tetramers. The numbers in the upper right quadrants indicate the frequency of NP396-specific CD8^+^ T cells within the total CD8^+^ T cell population in peripheral blood and represent the mean ± SD of three to five mice per group and time point.
(B–D) All mice were killed 7 d after challenge, and virus titers in liver, spleen, and blood were determined by immunofocus assay. Three additional naïve mice without LCMV-WE challenge infection were included as negative controls for the assay.
(E and F) On day 7, serum AST and ALT levels were determined. Four naïve mouse sera were tested in parallel and are shown for comparison.
(G) Liver tissue was collected from the animals killed on day 7. Sections were stained with H-E, and the severity of hepatitis was scored. The symbols in (B–G) represent individual mice. Statistical analysis was carried out for (E–G). Significant differences between groups are indicated as for *P < 0.05 or **P < 0.01.
(594 KB EPS)

Figure S6 Generation and Molecular Characterization of rLCMV-ARM* (A) Schematic describing the protocol for generation of rLCMV-ARM*.
(B): pS*(−) was designed analogous to pS_IND_ (also referred to as pSr(−) [36]). It expresses in genomic (−) polarity a wild-type LCMV S segment with two noncoding single nucleotide tags (*). Transcription is driven by the murine polymerase I promotor (PIP) and is terminated upstream of the murine polymerase I terminator (PIT). UTR, untranslated region; IGR, intergenic region.
(C and D) Genetic tags introduced at position 819 and 1467 of the NP ORF (with respect to the adenine in the start codon of the NP ORF as nucleotide 1) convert single C residues of the LCMV-ARM NP cDNA to T as in LCMV-WE without affecting the viral translation product. RT-PCR products with gene specific primers NP2743r plus NP2223f or Sseq5 plus Sseq2 span the respective tags allowing differentiation of wild-type and cDNA derived viral genomes by digestion with EcoNI and BbsI, respectively. Note that rLCMV/INDG should not yield an RT-PCR product with the Sseq5/Sseq2 primer pair due to the lack of binding of primer Sseq2 in the LCMV-GP ORF (C). Primers GP759r and GP247 detect the LCMV-GP ORF by RT-PCR. VSVGPf and VSVGPr primers amplify a fragment of INDG cDNA (not shown in the schematic).
(D) Total cellular RNA was collected from BHK-21 cells that had been infected with the indicated viruses for 48 h at a multiplicity of infection of 0.1. The different primer pairs were used to detect specific viral sequences as outlined in (C). All PCRs were carried out with (+RT) or without (−-RT) RT of the template to rule out the possibility that residual plasmid contaminations in rLCMV-ARM* served as template for the reactions. Arrow indicates the amplification products of the expected sizes. Products spanning the genetic tags are shown with or without prior digestion with BbsI or EcoNI, respectively. Considering complete digestion of the LCMV-ARM–derived PCR product by EcoNI, incomplete digestion by BbsI most likely resulted from an inhibitory effect of PCR buffer that had been present in the digestion reaction. Predicted fragment sizes are for BbsI digestion: 595 and 411 nucleotides; for EcoNI digestion: 269 and 251 nucleotides. Undigested PCR products of the expected sizes are indicated with arrows. Double-headed arrows indicate restriction fragments of the expected sizes.
(E) BHK-21 cells (10^6^ cells in M6 well) were infected with the indicated dose combination of LCMV-ARM and rLCMV/INDG for 48 h. Total cellular RNA was collected and rLCMV/INDG RNA was detected by RT-PCR as in (D). An arrow indicates the amplification product of the expected size.
(1.1MB EPS).

Figure S7 Indistinguishable CTL Response to LCMV-ARM and rLCMV-ARM* Infection (A) C57BL/6 mice were infected i.v. with 10^5^ PFU LCMV-ARM or rLCMV-ARM* and were killed on day 9 (same experiment as in Figure 5C). The frequency of GP33- and of NP396-specific CD8^+^ T cells in the spleen of mice was determined by MHC class I tetramer staining. Representative FACS plots of three mice per group are shown [except for a single naive control mouse (none)]. Numbers in the upper right quadrants indicate the percentage of epitope-specific CD8^+^ T cells within the total CD8^+^ T cell population (mean ± SD, except for naive control).
(B and C) GP33-specific (B) and NP396-specific (C) cytolytic activity of the same splenocytes as tested in (A). Symbols represent the mean ± SD of three mice per group (except for the single naïve control). (B and C) One representative of two independent experiments.
(408 KB EPS)

Figure S8 Inability of rLCMV/INDG to Establish Persistence in the Central Nervous System of Adult Immunocompetent Mice C57BL/6 mice were inoculated with 3 × 10^3^ PFU rLCMV/INDG intracerebrally, either in adulthood (“adult”) or within 24 h after birth (“neonatal”). Two weeks later, all animals were killed and brain RNA was processed for RT-PCR analysis. An NP-specific amplification product was obtained using the primers NP2223f and NP2743r, as described in Materials and Methods. Each lane represents an individual mouse.
(400 KB EPS)

Protocol S1 Supplementary Text Explanatory text to Figure S1 (Rapid Elimination of Intracerebral LCMV-ARM Infection by rLCMV/INDG-Induced Memory CTL), Figure S3 (rLCMV/INDG-Induced Immune Protection Is Rapidly Established), Figure S4 (Rapid and Potent nAb Response to rLCMV/NJG Infection), Figure S6 (Generation and Molecular Characterization of rLCMV-ARM*), and Figure S7 (Indistinguishable CTL Response to LCMV-ARM and rLCMV-ARM* Infection), with supplementary references and supplementary methods.
(80 KB DOC)

Table S1 Serological Characterization of Wild-Type and Recombinant LCM Viruses (36 KB DOC)

### Accession Numbers

The GenBank (http://www.ncbi.nlm.nih.gov/Genbank) accession numbers for the complete sequences of the cDNA-derived recombinant S segments in rLCMV/INDG, rLCMV/NJG, and rLCMV-ARM* are DQ408670, DQ408671, and DQ458914, respectively.

## Abbreviations

AGR: mice deficient in RAG as well as in type I and type II interferon receptors
ALT: alanine aminotransferase
AR: mice deficient in RAG as well as in type I interferon receptor
AST: aspartate aminotransferase
CTL: cytotoxic T cell
GP: glycoprotein
i.c: intracerebral(ly)
i.v: intravenous(ly)
INDG: VSV serotype Indiana GP
LCM: lymphocytic choriomeningitis
LCMV: lymphocytic choriomeningitis virus
LCMV-ARM: LCMVwt strain Armstrong
LF: Lassa fever
LFV: Lassa fever virus
mAb: monoclonal antibody
MV: Mopeia virus
nAb: neutralizing antibody
NJG: VSV serotype New Jersey glycoprotein
NP: nucleoprotein
NP396: immunodominant H-2D^b^−restricted LCMV-NP−derived CTL epitope
PFU: plaque-forming unit
rLCMV-ARM*: genetically engineered recombinant LCM virus of wild-type sequence modified by only two genetic tags
rLCMV/INDG: recombinant LCMV expressing INDG instead of LCMV-GP
rLCMV/NJG: recombinant LCMV expressing NJG instead of LCMV-GP
SN: supernatant
VSV: vesicular stomatitis virus
