# Supplementary material for: Envelope Exchange for the Generation of Live-Attenuated Arenavirus Vaccines
Source: PLoS Pathog. 2006 Jun 2;2(6):e51. doi: 10.1371/journal.ppat.0020051 (PMC1472708; doi:10.1371/journal.ppat.0020051)
Supplement: Supplementary file 1 [file ppat.0020051.sd001.doc]

**Protocol S1**

**Supplementary text**

**Rapid elimination of intracerebral LCMV-ARM infection by rLCMV/INDG induced memory CTL (compare to Fig. S1)**

We aimed to investigate the mechanisms of immune protection operating in rLCMV/INDG-immune mice challenged with LCMV-ARM i.c.. For LCMVwt immune mice it is known that protection against lethal LCM is mediated by memory CD8^+^ T cells (63, 64). In a representative experiment carried out 264 days after rLCMV/INDG immunization we therefore used MHC class I tetramer complexes to study the CTL recall response of rLCMV/INDG immune mice. Our analysis focused on CD8^+^ T cells specific for the immunodominant LCMV-NP derived epitope NP396, the major cytotoxic T cell (CTL) determinant shared between the immunizing rLCMV/INDG virus and LCMVwt strains such as the challenge virus LCMV-ARM. In mice immunized with rLCMV/INDG i.c. 264 days previously, about 0.3% NP396-specific memory CTL were found in the peripheral CD8^+^ T cell pool (Fig. S1A). Within four days after i.c. challenge with LCMV-ARM these cells expanded however to a frequency of about 10% while NP396-specific CTL were not yet detectable in previously naïve control mice (mean <0.1%). This rapid CD8^+^ recall response in rLCMV/INDG immune mice cleared LCMV-ARM from brain and spleen within five days after challenge (Fig. S1B), while viral titers in previously naïve mice were high at that time point. Thereby, LCMV-ARM clearance in rLCMV/INDG immune mice was apparently accomplished in a clinically silent manner before infection became too widespread, whereas the delayed CTL response in controls would have caused lethal immunopathology at around six days after infection (compare with Fig. 2C, Tbl. I).

**rLCMV/INDG-induced immune protection is rapidly established (compare to Fig. S3)**

Longevity of protection will be a key requirement for a vaccine to be routinely administered in endemic areas. In contrast, for health care workers or military personnel deployed to such places a vaccine should primarily provide rapid protection. The same criterion would also apply in case of a bioterrorist attack. Thus we tested how rapidly rLCMV/INDG immunization could confer protection against overwhelming LCMV-WE infection. Mice were immunized i.v. with rLCMV/INDG either six or only three days prior to high dose i.v. challenge with LCMV-WE (2x10^5^ PFU). Eight days later we determined virus titers in blood, spleen and liver, and we measured AST and ALT activity in the serum (Fig. S3). Mice that had been immunized with rLCMV/INDG either six or three days prior to LCMV-WE challenge controlled the challenge infection at or below the detection limit of our assay, whereas control mice without immunization exhibited high viral load in all organs tested (Fig. S3A-C, F-H). In accordance with these findings, strongly elevated AST and ALT levels were measured in non-immune mice challenged with LCMV-WE but not in either of the two immunized groups (Fig. S3D-E, I-J). Thus, rLCMV/INDG-induced protection against LCMV-WE-induced disease was established within only three days after immunization.

**Rapid and potent nAb response to rLCMV/NJG infection (compare to Fig. S4)**

We had previously observed that unlike for LCMVwt strains (31, 65) or LFV (9, 11, 32), rLCMV/INDG induced a rapid and potent neutralizing B cell response (31). To determine the capacity of rLCMV/NJG to elicit nAbs, C57BL/6 mice were infected with 2x10^4^ PFU of either LCMV-ARM, rLCMV/INDG, rLCMV/NJG, VSV-IND or VSV-NJ i.v. Serum samples collected at various time points were tested for their ability to neutralize VSV-IND or VSV-NJ in a plaque reduction assay (Fig. S4). VSV-NJ neutralizing serum activity, putatively IgM, was detectable in the serum of VSV-NJ or rLCMV/NJG infected mice as early as two days after infection (Fig. S4A). Neutralizing IgG appeared between day 4 and day 6 (Fig. S4B). These serum antibodies reached high titers and were maintained at substantial levels for at least 180 days after a single immunization. Analogous results were obtained with sera from rLCMV/INDG or VSV-IND infected mice tested for their neutralizing activity against VSV-IND (Fig. S4C,D). As expected, neutralization was strictly serotype specific with a complete lack of VSV-IND neutralizing activity in VSV-NJ or rLCMV/NJG immune sera and vice versa. Similarly, LCMV-ARM infection elicited neither nAbs against VSV-IND nor against VSV-NJ. These findings extended our previous conclusion that the viral surface antigen (LCMV-GP versus NJG or INDG) was the major limiting factor for virus nAb kinetics in LCMV infection (31). In the context of vaccination, this was of particular importance because it suggested that GP exchange rendered rLCMV/INDG as well as rLCMV/NJG efficiently controllable by nAbs (see also Discussion section).

**Generation and molecular characterization of rLCMV-ARM* (compare to Fig. S6)**

A schematic of the recovery protocol for rLCMV-ARM* is given in Fig. S6A. In analogy to the strategy used to recover rLCMV/NJG (compare to Fig. 4A) we transfected BHK-21 cells with a polymerase I (pol-I) driven vector for intracellular expression of a LCMV S segment RNA (Fig. S6B) in combination with polymerase II (pol-II) driven plasmids for coexpression of the minimal viral transacting factors NP and L (pC-NP, pC-L (2, 36)). 48 h later the transfected cells were infected with rLCMV/INDG helper virus. Supernatants harvested 12, 24 or 48 h later were propagated on duplicate cultures of fresh BHK-21 cell monolayers. The cells were fixed 24 h later and were stained for LCMV-GP and INDG respectively to assess in a semi-quantitative manner the amount of new reassortant rLCMV-ARM* but also of rLCMV/INDG helper virus (analogously to Fig. 4C, not shown). Immunofluorescence (IF) revealed only background levels of infectivity at 12 hours after infection followed by a continuous increase of both viruses with time. We decided to use supernatant harvested 24 h after rLCMV/INDG infection (P0/24h), containing a low but clearly detectable fraction of rLCMV-ARM* for further passage on fresh BHK-21 cell monolayers. INDG neutralizing monoclonal antibody (mAb) VI-7 (66) was added to the culture after an adsorption period of 90 min to select against rLCMV/INDG helper virus. Supernatant harvested 48 h later contained >10^6^ PFU of total LCMV infectivity (rLCMV-ARM* and rLCMV/INDG together, as assessed by standard immunofocus assay detecting LCMV-NP) and was directly used for plaque purification. LCMV-ARM but not rLCMV/INDG forms lytic plaques on VERO cells under standard conditions (36) rendering the isolation of clonal rLCMV-ARM populations an easy procedure (not shown).

To differentiate cDNA derived S segments from naturally occuring LCMV strains, all S segment cDNAs (including rLCMV/INDG) carry two non-coding mutations in the NP ORF in order to distinguish them from LCMVwt (Fig. 1A and S6B, C). These tags were designed to convert single nucleotides in ARM-NP to the corresponding position of the closely related WE strain of LCMV. Thereby, we avoided at best any detrimental effects of mutagenesis on yet unknown but potentially important secondary RNA structures in the NP ORF. Single nucleotide transitions mutated the BbsI and EcoNI recognition motives in the NP cDNA (Fig. S6C) and allowed the differentiation of rLCMV-ARM* and LCMV-ARM by restriction digestion of RT-PCR products spanning the respective sequence stretches (Fig. S6D). As predicted, RT dependent NP specific PCR products of the latter but not of the former virus could be digested with BbsI or EcoNI, respectively. This confirmed the cDNA origin of the rLCMV-ARM* S segment (Fig. S6D). The precise nucleotide sequences of the rLCMV-ARM* derived RT dependent PCR products were obtained after cloning in a T/A-vector and supported our conclusion (not shown). Further characterization of the RNA profile indicated that rLCMV-ARM* and LCMV-ARM but not rLCMV/INDG expressed LCMV-GP RNA. As expected, INDG RNA was amplified from rLCMV/INDG infected cells while rLCMV-ARM* yielded no signal (Fig. S6D). A single PFU of rLCMV/INDG added to an inoculum of 10^4^ PFU LCMV-ARM was however readily detected by INDG specific RT-PCR (Fig. S6E), indicating that the RT-PCR protocol used would have been sufficiently sensitive to detect even minor contamination of remaining helper virus in rLCMV-ARM*.

**Indistinguishable CTL response to LCMV-ARM and rLCMV-ARM* infection (compare to Fig. S7)**

We had observed that the virus load in spleen and liver of mice infected with rLCMV-ARM* or LCMV-ARM was indistinguishable, indicating that the two viruses were of equivalent fitness (Fig. 5C). As an additional indirect readout for the viral burden in rLCMV-ARM*-infected mice, the frequencies of CD8^+^ T cells specific for the immunodominant H-2^b^ restricted epitopes NP396 and GP33 were determined by MHC class I tetramer staining and were found to be indistinguishable from LCMV-ARM-infected mice (Fig. S7A). To test the effector function of rLCMV-ARM* induced CTLs, virus-specific cytolytic activity of splenocytes was measured in a primary ex vivo Cr^51^ release assay (Fig. S7B, C). Unlike rLCMV/INDG infection eliciting very low primary CTL activity (31), rLCMV-ARM*-induced CD8^+^ effector T cells exhibited equally high cytolytic capacity than those of LCMV-ARM infected mice, corroborating that the two viruses were phenotypically indistinguishable.

**Supplementary references**

63.	Cole, G.A., N. Nathanson, and R.A. Prendergast. 1972. Requirement for theta-bearing cells in lymphocytic choriomeningitis virus-induced central nervous system disease. *Nature* 238:335-337.

64.	Cole, G.A. 1986. Production or prevention of neurologic disease by continuous lines of arenavirus-specific cytotoxic T lymphocytes. *Med Microbiol Immunol (Berl)* 175:197-199.

65.	Seiler, P., M.A. Brundler, C. Zimmermann, D. Weibel, M. Bruns, H. Hengartner, and R.M. Zinkernagel. 1998. Induction of protective cytotoxic T cell responses in the presence of high titers of virus-neutralizing antibodies: implications for passive and active immunization. *J Exp Med* 187:649-654.

66.	Kalinke, U., E.M. Bucher, B. Ernst, A. Oxenius, H.P. Roost, S. Geley, R. Kofler, R.M. Zinkernagel, and H. Hengartner. 1996. The role of somatic mutation in the generation of the protective humoral immune response against vesicular stomatitis virus. *Immunity* 5:639-652.

67.	Battegay, M., D. Moskophidis, H. Waldner, M.A. Brundler, W.P. Fung-Leung, T.W. Mak, H. Hengartner, and R.M. Zinkernagel. 1993. Impairment and delay of neutralizing antiviral antibody responses by virus-specific cytotoxic T cells. *J Immunol* 151:5408-5415.

68.	Charan, S., and R.M. Zinkernagel. 1986. Antibody mediated suppression of secondary IgM response in nude mice against vesicular stomatitis virus. *J Immunol* 136:3057-3061.

69.	Scott, D.W., and R.K. Gershon. 1970. Determination of total and merecaptothanol-resistant antibody in the same serum sample. *Clin Exp Immunol* 6:313-316.

70.	Gossmann, J., J. Lohler, and F. Lehmann-Grube. 1991. Entry of antivirally active T lymphocytes into the thymus of virus-infected mice. *J Immunol* 146:293-297.

**Supplementary methods**

### RNA analysis by RT-PCR and restriction digestion

Total cellular RNA was extracted using TriReagent (Molecular Research Center). RT with random hexamer primers was done using Superscript II (Roche) according to the manufacturers instructions. Gene specific products were obtained upon PCR amplification with Taq polymerase (Roche) using the following primer pairs. NP2743r 5’- CAATGACGTTGTACAAGCGC-3’ and NP2223f 5’- GCATTGTCTGGCTGTAGCTTA-3’ yielded a product of 520nt spanning the EcoNI site in LCMV-ARM NP. Digestion with EcoNI yielded two fragments of 269 and 251 nt. Sseq5 5’- ACCTCAGGTGGGCTTAAGCTA-3’ and Sseq2 5’- GGCTTGTCACCAATGGTTC-3’ amplified a 1006nt stretch spanning the BbsI site in LCMV-ARM NP. Digestion with BbsI yielded 595 and 411nt fragments. Primers GP759r 5’- GGTATTGGTAACTCGTCTGGC-3’ and GP247f 5’- GTGGCATGTACGGTCTTAAGG-3’ amplified a 512 nt fragment of LCMV-GP. INDG was detected using VSVGPf 5’-GTCCATAACTCTACAACCTGGC–3’ and VSVGPr 5’- CGACTCTGATGTATCTGGTCTC–3’ giving a 519nt product. Amplification of a 746nt NJG fragment was done with primers NJGf 5’-CCTCCTCAGAGTTGTGGGTATGG–3’ and NJGr 5’-CCGCACAATCTTAGTTCCTGC-3’. Precise individual PCR conditions are available from the authors upon request.

## Cytotoxicity assay

Virus specific cytotoxic activity of spleen cells was assayed as described previously (40). Briefly, single-cell suspensions were prepared from the spleens of mice at day 9 after infection and were used directly in a primary ex vivo ^51^Cr release assay. Target cells were EL-4 cells coated with GP33-41 or NP396-404 (10^-6^M) and uncoated control cells. Background killing on uncoated control target cells was insignificant in all experiments shown and was subtracted to obtain specific lysis (%). The immunodominant H-2D^b^ binding LCMV peptides gp33-41 (GP33) and np396-404 (NP396) were purchased from Neosystem Laboratoire (Strasbourg, France).

### Plasmids

Plasmids pC-L and pC-NP expressing the LCMV L and NP proteins under control of polymerase II have been described (2, 36). pS*(-) and pS_NJ_(-) were generated by a cloning strategy previously outlined in detail (36). Briefly, the LCMV-GP ORF was amplified from pC-LCMV-GP (2) using PFU polymerase (Stratagene) and primers 5’-AAT**CGTCTC**T*AAGG*ATGGGTCAGATTGTGACAATG-3’ and 5’-AAT**CGTCTC**T*TTCT*TCAGCGTCTTTTCCAGACGG-3’ with a 5’ flanking AAT overhang for facilitated digestion of the BsmBI sites (bold), a spacing T and four nucleotides from the UTR and IGR, respectively (italic) followed downstream by the LCMV-GP N- and C-terminal sequences, respectively. The PCR product was digested with BsmBI and inserted into the equally prepared pSBsm(-) vector (36) to reconstitute a full length LCMV S segment cDNA under control of the murine polymerase I promotor (see Fig. S4B). For generation of a VSV-NJG cDNA, total RNA was collected from VSV-NJ infected cells as described above and the NJG sequence was amplified using primers 5‘-AAT**CGTCTC**T*AAGG*ATGTTGTCTTATCTAATCTTTGC-3’ and 5’-AAT**CGTCTC**T*TTCT*TTAACGGAAATGAGCCAT-3’ with 5’ flanking sequences as described above. The PCR product was digested with BsmBI and directly inserted into the equally prepared acceptor cassette of pSBsm(-) to create pS_NJ_(-).

**Monoclonal antibodies, hyperimmune serum and neutralization assays**

mAbs that had been generated by immunizing mice with LCMV-WE (WEN-3), VSV-IND (VI-7) and VSV-NJ (H6B9D5) have been described (65, 66). LCMV-ARM hyperimmune sera (HIS) were generated as described previously (31). Neutralizing antibodies (nAbs) against LCMV and rLCMV/INDG were detected in a focus reduction assay as previously described for LCMV (67). VSV nAbs were measured by standard plaque reduction assays (68). VSV neutralizing IgG was determined after inactivation of IgM by 2-mercaptoethanol (2-ME) (69). Despite controversies over the specificity of this procedure it remains the only method of determining virus neutralizing IgG reliably in a plaque reduction assay because specific secondary antibodies cannot be used. Total neutralizing serum antibody concentrations exceeding IgG by two or more titer steps were considered to be IgM.

### Immunofluorescence

Tissue was collected as described for histopathological scoring, and immunohistochemistry was performed after unmasking of antigen by microwave treatment (15 min., 800W) in citrate buffer. Sections were blocked with 10% fetal calf serum in PBS for 10 min at RT. Washed sections were stained with the following primary antibodies: mouse anti-human CD3 (Serotec, Germany) and with rabbit anti-LCMV polyclonal serum (70). Bound antibody was visualized with Cy3- or Cy2-conjugated goat-anti rabbit IgG and donkey anti-rat IgG (both from Jackson, ImmunoResearch). Nuclei were visualized with DAPI staining (Sigma).
