# Supplementary material for: Envelope Exchange for the Generation of Live-Attenuated Arenavirus Vaccines
Source: PLoS Pathog. 2006 Jun 2;2(6):e51. doi: 10.1371/journal.ppat.0020051 (PMC1472708; doi:10.1371/journal.ppat.0020051)
Supplement: Supplementary file 2 [file ppat.0020051.st001.doc]

Tbl. SI: Serological characterization of wild type and recombinant LCM viruses

|  | Experiment 1 | | Experiment 2 | | |
| --- | --- | --- | --- | --- | --- |
| Antibody/ serum ^A^ | LCMV-  ARM ^2^ | rLCMV  -ARM* ^B^ | LCMV-  ARM ^B^ | rLCMV/  INDG ^B^ | rLCMV/  NJG ^B^ |
| WEN-3 ^C^ | 6 | 6 | 5 | <1 | <1 |
| VI-7 ^C^ | <1 | <1 | <1 | >12 | <1 |
| H6B9D5 ^C^ | n.t. | n.t. | <1 | <1 | >12 |
| LCMV-ARM HIS ^C^ | 3 | 3 | 4 | <1 | <1 |
| LCMV-ARM i.v. d12 ^D^ | n.t. | n.t. | <1 | <1 | <1 |
| VSV-IND i.v. d12 ^D^ | n.t. | n.t. | <1 | 9 | <1 |
| VSV-NJ i.v. d12 ^D^ | n.t. | n.t. | <1 | <1 | 9 |

^A^	Monoclonal antibodies and immune mouse sera were tested in immunofocus reduction assays for virus neutralizing activity. Neutralizing titers were determined for 10-fold prediluted sera and the values obtained are represented as (-log_2_). Two independent experiments (1 and 2) are summarized; n.t.: not tested

^B^	Virus used for immunofocus reduction assays.

^C^	LCMV-ARM hyperimmune serum (HIS), mAb WEN-3 (anti-LCMV), mAb VI-7 (anti-VSV-IND) and mAb H6B9D5 (anti-VSV-NJ) are described in the Supplementary Methods section.

^D^	C57BL/6 mouse serum was collected 12 days after i.v. infection with 2x10^4^ PFU of either of the indicated viruses (same mice as in Fig. S6). Note that LCMV-ARM infection is well known not to elicit detectable virus neutralizing serum activity for >60 days (30, 55)!
